# A Post-Processing Method for Quantum Random Number Generator Based on Zero-Phase Component Analysis Whitening

**DOI:** 10.3390/e27010068

**Published:** 2025-01-14

**Authors:** Longju Liu, Jie Yang, Mei Wu, Jinlu Liu, Wei Huang, Yang Li, Bingjie Xu

**Affiliations:** National Key Laboratory of Security Communication, Institute of Southwestern Communication, Chengdu 610041, China; longju_liu@163.com (L.L.); wumei_tyut@163.com (M.W.); sunny4work@163.com (J.L.); huangwei096505@aliyun.com (W.H.); yishuihanly@pku.edu.cn (Y.L.)

**Keywords:** quantum random number generator, post-processing, zero-phase component analysis whitening

## Abstract

Quantum Random Number Generators (QRNGs) have been theoretically proven to be able to generate completely unpredictable random sequences, and have important applications in many fields. However, the practical implementation of QRNG is always susceptible to the unwanted classical noise or device imperfections, which inevitably diminishes the quality of the generated random bits. It is necessary to perform the post-processing to extract the true quantum randomness contained in raw data generated by the entropy source of QRNG. In this work, a novel post-processing method for QRNG based on Zero-phase Component Analysis (ZCA) whitening is proposed and experimentally verified through both time and spectral domain analysis, which can effectively reduce auto-correlations and flatten the spectrum of the raw data, and enhance the random number generation rate of QRNG. Furthermore, the randomness extraction is performed after ZCA whitening, after which the final random bits can pass the NIST test.

## 1. Introduction

Random numbers (RNs) are widely used in many fields, such as games, simulations, and cryptography [1]. In the field of cryptography and information security, reliable generation of unpredictable random sequences is an important issue. Depending on the means of generation, random numbers can be divided into two main types: pseudo-random numbers and true random numbers. Pseudo-random numbers are statistically random sequences generated using deterministic algorithms whose randomness depends on the initial seed and the mathematical algorithm, and thus do not achieve true randomness [2]. Quantum Random Number Generators (QRNGs) are a type of true random number generator that exploit the inherent randomness results from quantum processes to create random numbers with informational provable security [3].

In recent years, many QRNG schemes have been proposed, and significant progress has been achieved [4]. For example, one important scheme based on the detection of single photon’s path choice after a 50:50 beam splitter has been implemented [5,6]. Another significant scheme based on single photon detection is to measure the arrival times of photons on a photodetector, which has been studied and achieves a higher generation rate [7,8,9]. However, due to the dead time of single photon detector (SPD), the generation rate of the two schemes are limited. To achieve a higher generation rate, QRNG schemes based on macroscopic detection are proposed by researchers, including measuring the vacuum fluctuations [10,11,12,13,14], the quantum phase noise [15,16,17,18,19], and the amplified spontaneous emission noise [20,21,22]. The output of the quantum entropy sources of these schemes is continuous, and the advantage is that the generation rate is very high, reaching 100 Gbps level [14,19,22,23].

**Figure 1:**
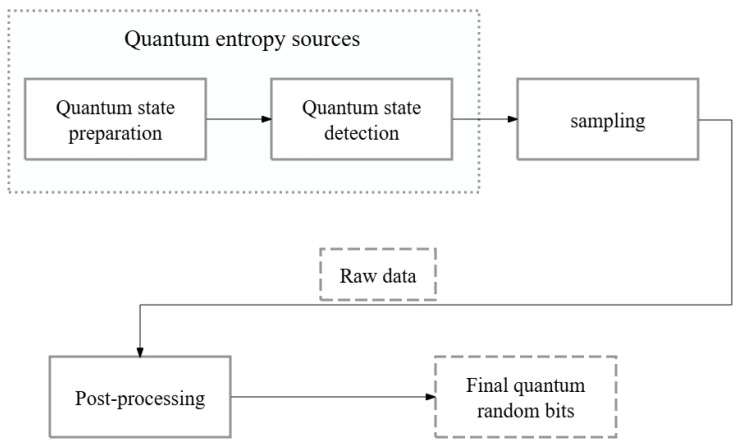
The typical structure of a QRNG.

Generally, the typical structure of a QRNG is shown as Figure 1, which consists of the quantum entropy source (QES), sampling, and post-processing. The QES usually includes the quantum state preparation, quantum state detection, then the sampling is performed which can generate the raw data sequence. Due to the classical noise and device imperfections of the QRNG system, the raw data contains bias and correlation inevitably. In order to obtain better randomness, it is necessary to perform post-processing. Many approaches for post-processing have been proposed and demonstrated, such as the Von Neumann method and the universal hash function [24].

In general, to avoid oversampling, which leads to high auto-correlation and reduced randomness of the raw data, the sampling rate in the experiment cannot significantly exceed the bandwidth of the QES, which is generally limited to within a maximum sampling rate of twice the bandwidth of the QES. The final random sequence generation rate is proportional to the sampling rate, so the final random sequence generation rate can be severely affected by the bandwidth of the QES. Throughout the current post-processing methods, there has never been a way to enhance the bandwidth of the QES. After the raw data are acquired, it may be possible to perform certain operations on raw data to increase the bandwidth of the signal before the entropy evaluation, such as the whitening process.

Zero-phase Component Analysis (ZCA) whitening is a fundamental preprocessing technique widely adopted in statistics and machine learning for removing feature correlations while standardizing their variances to unity [25]. Unlike Principal Component Analysis (PCA) whitening, which aligns data along eigenvectors and may distort the spatial arrangement, ZCA whitening employs a symmetric whitening matrix. This symmetry ensures minimal transformation distortion, preserving the visual or structural resemblance between the original and whitened data. Such characteristics make ZCA whitening particularly suitable in tasks like image recognition, signal processing, and biological data analysis, where preserving structural fidelity is critical for downstream interpretation and analysis.

Empirical studies have demonstrated that ZCA whitening minimizes the total squared distance between the raw and whitened data, achieving maximal similarity while effectively simplifying feature representations [25,26,27]. This approach uniquely balances the competing goals of efficient preprocessing and data integrity preservation, addressing a fundamental need in many scientific and engineering disciplines. Moreover, ZCA whitening is often preferred in scenarios where interpretability and minimal adjustment are essential, such as variable selection, feature extraction, and high-dimensional data analysis.

In comparison to other whitening methods, by employing the ZCA whitening method, it is feasible to retain the original coordinate system, which offers a clear advantage in applications demanding both accuracy and structural preservation. Overall, the simplicity, efficiency, and fidelity of ZCA whitening position it as a cornerstone preprocessing technique across diverse fields.

In this paper, we propose a post-processing method for QRNG based on ZCA whitening before randomness extraction. By performing a simple ZCA whitening on the raw data, the processed data have much lower auto-correlation and flatter power spectral density, which ensures that there is approximately no linear relationship between the processed data and the 3 dB bandwidth of the QES is correspondingly enhanced simultaneously. Based on the proposed method, the sampling rate of the QES can be significantly increased, which can enhance the Quantum Random Number Generation rate for QRNG.

Unlike traditional post-processing methods that primarily focus on randomness extraction to eliminate bias and correlations, this ZCA-whitening-based method is applied at the raw data stage, aiming to optimize the random data itself. This optimized data provides a better input for randomness extraction algorithms, ultimately improving their efficiency and effectiveness. It is worth noting that ZCA whitening does not replace randomness extraction algorithms directly. Instead, it functions as a complementary data preprocessing step, optimizing the input data to make the randomness generation process more effective.

To the best of our knowledge, this study is the first to propose a data processing approach for optimizing raw random sequences prior to randomness extraction in QRNG. While previous research has focused on post-processing, the optimization of raw random data has been largely overlooked. This approach provides a novel framework to improve the quality and efficiency of randomness generation.

## 2. Methods

**Figure 2:**
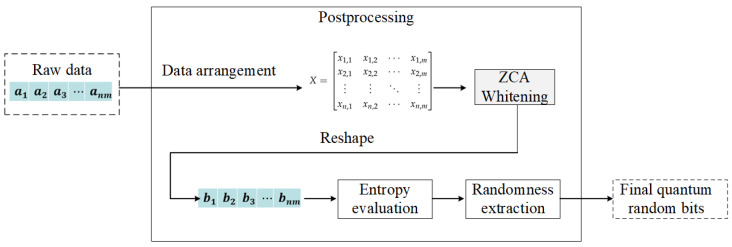
The proposed post-processing scheme for QRNG.

The proposed post-processing method for QRNG based on ZCA whitening method is shown in Figure 2. Firstly, the data matrix is constructed by arranging the raw data $A=\left\{a_{1},a_{2},a_{3},\cdots ,a_{nm}\right\}$. Each element $a_{i}$ of *A* is a real number ($a_{i}\in R$), acquired from the Quantum Random Number Generation (QRNG) process. These values originate from the amplitude of quantum noise and exhibit a statistical distribution determined by the physical properties of the quantum entropy source. Then, ZCA whitening is performed on these data matrix to obtain a processed data matrix. Next, this new data matrix is reshaped into a one-dimensional time sequence $B=\left\{b_{1},b_{2},b_{3},\cdots ,b_{nm}\right\}$. Finally, the entropy evaluation and randomness extraction is performed on the sequence *B*. More details of this post-processing scheme are shown as follows.

### 2.1. Principles of ZCA Whitening

ZCA whitening is an effective data processing method that aims to reduce the correlation of input data. Given an input data matrix, Zero-phase Component Analysis (ZCA) whitening is a preprocessing technique that removes correlations between data dimensions while preserving the original spatial structure of the data. Given a raw data matrix,

(1) $$X=\left[\begin{array}{cccc} x_{1,1} & x_{1,2} & \cdots & x_{1,m}\\ x_{2,1} & x_{2,2} & \cdots & x_{2,m}\\ \vdots & \vdots & \ddots & \vdots \\ x_{n,1} & x_{n,2} & \cdots & x_{n,m} \end{array}\right],$$

where $X\in R^{n\times m}$, the whitening process begins by centralizing the data. For each row i=1,2,⋯,n, the mean is computed as

(2) $${\bar{x}}_{i}=\frac{1}{m}\sum_{j=1}^{m}x_{i,j},$$

and a centralized data matrix is formed as

(3) $$X_{c}=X-\bar{X},$$

where $\bar{X}\in R^{n\times m}$ is the mean matrix, and every element in the *i*-th row of $\bar{X}$ is equal to ${\bar{x}}_{i}$.

The covariance matrix of the centralized data are computed as

(4) $$\Sigma =\frac{1}{m}X_{c}X_{c}^{T},$$

which is symmetric and positive semi-definite. To de-correlate the data, eigenvalue decomposition of the covariance matrix is performed, yielding

(5) $$\Sigma =U\Lambda U^{T},$$

where $U\in R^{n\times n}$ is an orthogonal matrix of eigenvectors, and $\Lambda =\mathrm{diag}(\lambda_{1},\lambda_{2},\cdots ,\lambda_{n})$ is a diagonal matrix containing the eigenvalues $\lambda_{i}\geq 0$. To ensure numerical stability, eigenvalues close to zero may be regularized by adding a small constant ϵ>0, resulting in

(6) $$\Lambda_{\mathrm{reg}}=\Lambda +\epsilon I.$$

The whitening matrix is constructed as

(7) $$W=U\Lambda^{-1/2}U^{T},$$

where

(8) $$\Lambda^{-1/2}=\mathrm{diag}\left(\lambda_{1}^{-1/2},\;\lambda_{2}^{-1/2},\;\cdots ,\;\lambda_{n}^{-1/2}\right).$$

Applying the whitening matrix to the centralized data yields the whitened data matrix,

(9) $$X_{zca}=WX_{c}.$$

After this transformation, the covariance matrix of $X_{zca}$ becomes the identity matrix,

(10) $$\Sigma_{zca}=\frac{1}{m}X_{zca}X_{zca}^{T}=I,$$

indicating that the dimensions are uncorrelated, and their variances are normalized to 1.

After ZCA whitening, the covariance of the data matrix becomes a unit matrix, which means that the correlation of different rows is eliminated, thus randomness of the input data are enhanced.

### 2.2. ZCA Whitening for QRNG

ZCA whitening is applied to the raw data generated by the quantum entropy source (QES) to enhance statistical randomness and reduce correlations. Let the raw data sequence be denoted as

(11) $$A=\{a_{1},a_{2},a_{3},\cdots ,a_{nm}\}.$$

**Figure 3:**
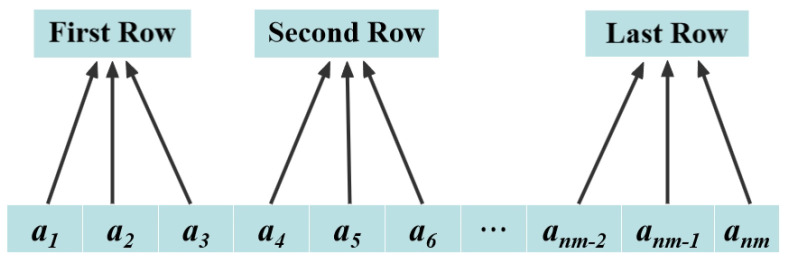
Data blocks when m=3.

Consider a matrix *X* of size n×m, where each element $a_{i}\in R$ represents a raw data point. A total of n×m raw samples are continuously collected and grouped into *n* data blocks, with each block containing *m* consecutive samples. The resulting matrix *X* consists of *n* rows and *m* columns, where each row corresponds to a data block formed by *m* consecutive raw samples. Figure 3 represents the data blocks in the time series when m=3.

The raw data are reshaped into a matrix $X\in R^{n\times m}$ as follows:

(12) $$X=\left[\begin{array}{cccc} a_{1} & a_{2} & \cdots & a_{m}\\ a_{m+1} & a_{m+2} & \cdots & a_{2m}\\ \vdots & \vdots & \ddots & \vdots \\ a_{(n-1)m+1} & a_{(n-1)m+2} & \cdots & a_{nm} \end{array}\right].$$

To perform ZCA whitening, the centralized matrix $X_{c}$ is first obtained by subtracting the mean of each row from the respective elements, as described in Section 2.1. The covariance matrix of $X_{c}$ is then computed, followed by its eigenvalue decomposition. The whitening matrix *W* is constructed, and the ZCA-transformed matrix $X_{zca}$ is obtained using the whitening transformation, as described in Equation (9).

The ZCA transformation ensures that the rows of $X_{zca}$ are uncorrelated, with the covariance matrix becoming an identity matrix. After whitening, the matrix $X_{zca}\in R^{n\times m}$ is reshaped into a one-dimensional sequence $B=\{b_{1},b_{2},\ldots ,b_{nm}\}$, suitable for randomness extraction. The reshaping process is defined as follows:

(13) $$X_{zca}=\left[\begin{array}{cccc} b_{1} & b_{2} & \cdots & b_{m}\\ b_{m+1} & b_{m+2} & \cdots & b_{2m}\\ \vdots & \vdots & \ddots & \vdots \\ b_{(n-1)m+1} & b_{(n-1)m+2} & \cdots & b_{nm} \end{array}\right],$$

where the elements $b_{i}$ are taken sequentially from the rows of $X_{zca}$. Mathematically, the mapping from $X_{zca}$ to *B* is expressed as

(14) $$b_{(i-1)m+j}=X_{zca}\left(i,j\right),\;\mathrm{for}\;i=1,2,\ldots ,n\;\mathrm{and}\;j=1,2,\ldots ,m.$$

This ensures that the elements $b_{1},b_{2},\ldots ,b_{nm}$ preserve the original order of the data from $X_{zca}$.

We denote the centralized matrix $X_{c}$ as

(15) $$X_{c}=\left[\begin{array}{c} c_{1}\\ c_{2}\\ \vdots \\ c_{n} \end{array}\right].$$

Its covariance matrix is

(16) $$\Sigma =\frac{1}{m}X_{c}X_{c}^{T}=\frac{1}{m}\left[Cov(c_{i},c_{j})\right].$$

Due to the correlation between different rows, the non-diagonal elements of the covariance matrix are not 0.

After perform ZCA whitening transformation on matrix *X*, we obtain the matrix $X_{zca}$. Denote the matrix $X_{zca}$ as

(17) $$X_{zca}=\left[\begin{array}{c} d_{1}\\ d_{2}\\ \vdots \\ d_{n} \end{array}\right].$$

The calculation for the correlation coefficient between any two row vectors of the matrix $X_{zca}$ is expressed as

(18) $$\rho_{ij}=\frac{Cov(d_{i},d_{j})}{\sigma_{i}\sigma_{j}},$$

where $\sigma_{i}$ and $\sigma_{j}$ is the standard deviation of the row vectors $d_{i}$ and $d_{j}$, respectively.

As explained in the previous subsection, the covariance of the matrix $X_{zca}$ is expected to be a unit matrix (i.e., $Cov(c_{i},c_{j})=0$ given i≠j), which means that ZCA whitening can remove the correlation between different rows of the raw data matrix.

The ZCA whitening process effectively removes correlations between blocks of raw data (each block consisting of *m* consecutive samples in the time domain), thereby enhancing the statistical randomness of the data. Correlation between rows is eliminated by performing ZCA whitening, i.e., correlation between raw data blocks consisting of m consecutive data in the time domain is eliminated.

Notably, another way of arranging the data are that each column is a consecutive *n* data in the time domain, arranged sequentially by column.

## 3. Experimental Verification

**Figure 4:**
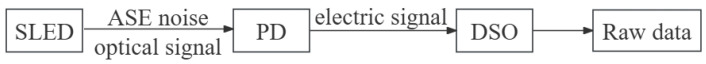
Experimental set up of ASE scheme to acquire the raw data. SLED: superluminescent light emitting diode; PD: photodetector; DSO: digital storage oscilloscope.

An QRNG based on amplified spontaneous emission (ASE) noise as shown in Figure 4 is implemented to verified the efficiency of the proposed ZCA whitening method. A SLED (EXSLOS, EXS210059-01) with center wavelength of 1550 nm is used to generate the ASE noise. Then, the ASE noise is detected by a 2 GHz PD, after which we use the digital storage oscilloscope (DSO, Keysight, DSOV084A) to sample the output signal of PD with sampling rate 10 GSa/s to acquire the raw data.

**Figure 5:**
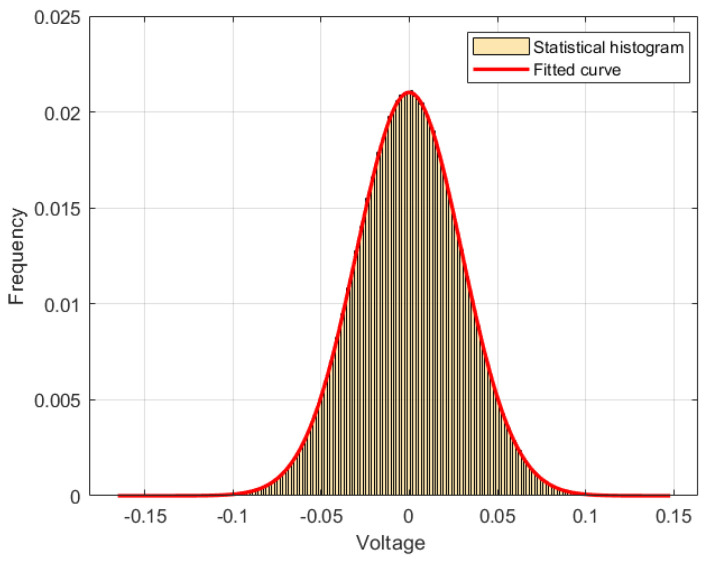
Statistical histogram of raw data for the QRNG based on ASE noise.

We choose the length of the raw data as $10^{6}$. Figure 5 shows the statistical histogram of raw data, which satisfy the Gaussian distribution. The raw data are arranged in order of rows according to the data matrix *X*, where each row contains consecutive $10^{3}$ data in the time domain; then, we have the data matrix $X\in R^{10^{3}\times 10^{3}}$.

**Figure 6:**
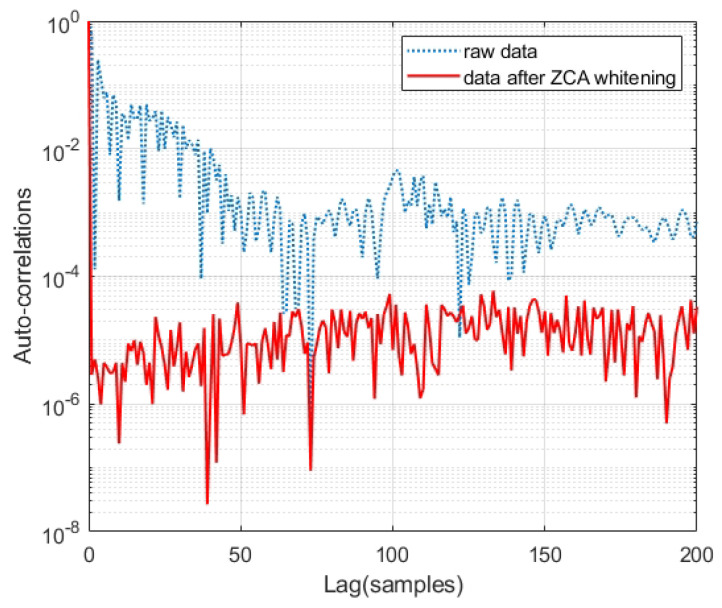
Auto-correlation coefficients before and after ZCA whitening.

Following the steps of algorithm presented above, the ZCA whitening is performed on the raw data matrix *X*. Then, we compared the auto-correlation coefficients of the raw data and the data after ZCA whitening. Figure 6 shows that the auto-correlation coefficient of the data after ZCA whitening is significantly lower. The average of the absolute values of the auto-correlation coefficient of the raw data are $1.03\times 10^{-2}$, and that of the data after ZCA whitening is $1.51\times 10^{-5}$.

**Figure 7:**
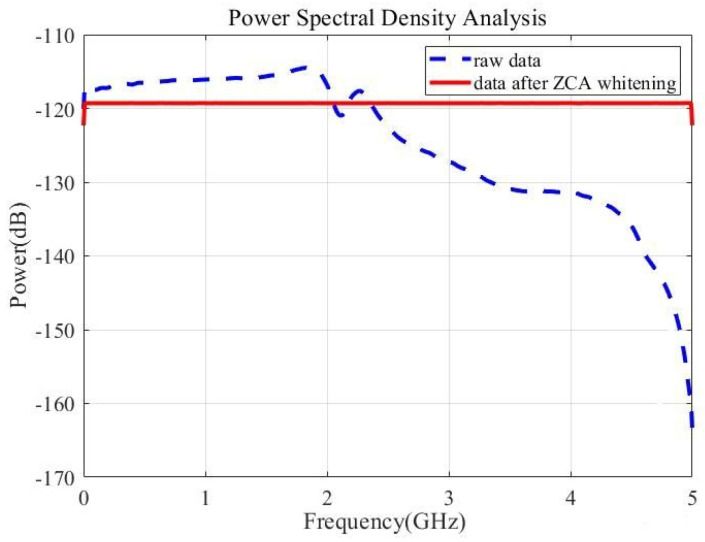
Power spectral density analysis before and after ZCA whitening.

The spectrum of the raw data and the data after ZCA whitening is shown in Figure 7. One can find that the data after whitening has a significantly flatter spectral curve with an enhanced 3 dB bandwidth compared to that of the raw data. In general, the sampling rate cannot significantly exceed the bandwidth of the QES. In this experiment, the sampling rate is 10 GSa/s, which is much larger than the QES bandwidth of 2 GHz, leading to a high auto-correlation coefficients shown as the blue curve in Figure 6. By performing the ZCA whitening on the raw data, the bandwidth is to some extent equalized to 5 GHz as shown in Figure 7, which means that the raw data can be in principle down sampled to 5 GSa/s and the random number generation rate can be increased.

Next, the entropy evaluation and randomness extraction is performed on the data after ZCA whitening. Min-entropy ($H_{min}$) measures the unpredictability of a system based on the most likely outcome. It is particularly significant in contexts where randomness or uncertainty plays a crucial role, such as random number generation, cryptography, and data compression. Unlike other entropy measures such as Shannon entropy, which considers the average uncertainty of all outcomes, min-entropy focuses purely on the most likely outcome. $H_{min}$ directly evaluates the amount of random bits that can be extracted from each raw data sample. Analytically, for a discrete random variable *X* with probability distribution $P=\{p_{1},p_{2},\ldots ,p_{n}\}$, the min-entropy is expressed as:

(19) $$H_{min}\left(P\right)=-\log\left(\max(p_{1},p_{2},\ldots ,p_{n})\right),$$

where $\max(p_{1},p_{2},\ldots ,p_{n})$ represents the highest probability of the outcomes $x_{1},x_{2},\ldots ,x_{n}$ in the distribution, and the logarithm base is typically 2.

We assume that the system is secure, and we consider a practical scenario where quantum attacks are not taken into account. The focus of our study is presently on validating the principles of the ZCA method in randomness generation, rather than analyzing the system’s performance under quantum side-channel attacks. Therefore, using classical min-entropy as the evaluation method is sufficient for the scope of this study. The min-entropy of the data after ZCA whitening is estimated to be 9.3327 per 12-bit sample. Then, the m-least-significant-bit (m-LSB) procedure and the bitwise exclusive OR (XOR) operation are employed for randomness extraction based on the entropy evaluation results. We reserve 8-LSBs from each sample after XOR operation to generate the final quantum random bit sequences, and the random number generation rate is 40 Gbps.

**Figure 8:**
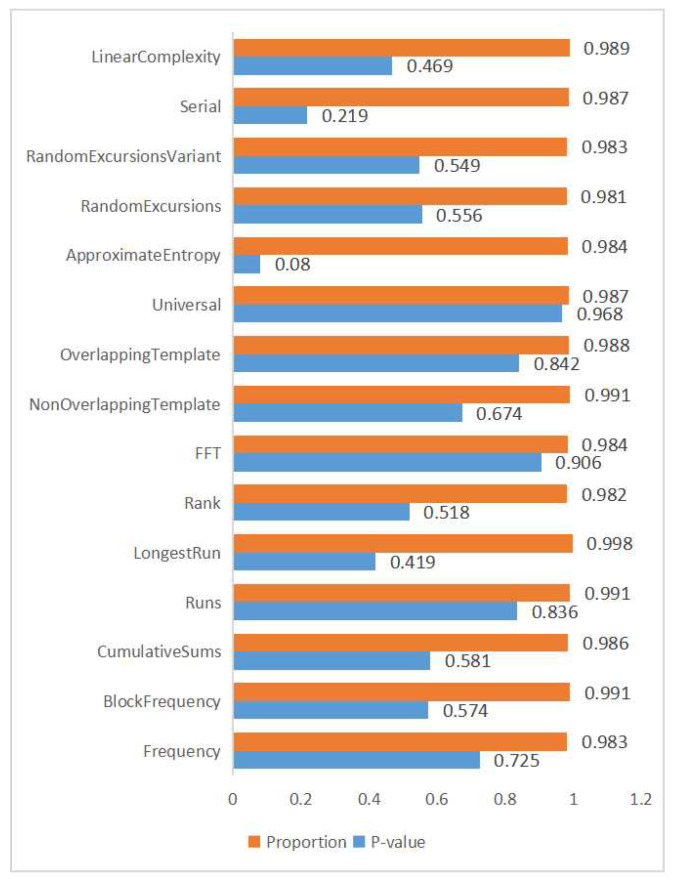
The results of the NIST-STS test.

The NIST Statistical Test Suite (NIST-STS) is a comprehensive set of statistical tests developed by the National Institute of Standards and Technology for evaluating the quality and randomness of binary sequences. It is widely used to verify the statistical properties of random number generators (RNGs), especially in cryptographic applications. The suite includes a total of 15 tests, such as the frequency test, block frequency test, cumulative sums test, and approximate entropy test, which collectively evaluate various aspects of randomness, including uniformity, independence, and unpredictability of sequences [28]. To assess the statistical properties of the generated random numbers, we employed the NIST Statistical Test Suite (NIST-STS) [28]. For a test to be considered passed, the *p*-value of the test statistic must satisfy p≥0.01, indicating that the observed sequence does not deviate significantly from randomness. Figure 8 shows the results of the NIST-STS test, and the *p*-value for each test is greater than 0.01, which indicates that the final random bits have passed all the NIST-STS tests, confirming the high quality and randomness of the random sequences.

The experiment results sufficiently demonstrate the effectiveness of post-processing method based on ZCA whitening in terms of enhancing the bandwidth of raw data. A potential physical explanation for this enhancement is described as follows. For a general high speed QRNG, the bandwidth of the quantum entropy source is directly limited by the detector, where the components in the frequency band within 3 dB bandwidth in principle dominate the signal in power and is extracted for random number generation. However, the signal components in the frequency band outside the 3 dB bandwidth of the spectrum also contain quantum randomness, but with less significant power. In this scenario, the ZCA whitening poses an approach to lower the power of the frequency band within 3 dB bandwidth, while enhancing the power of the counterpart outside of the 3 dB bandwidth, based on which the spectrum is flattened to achieve an equal enhancement for the entropy bandwidth where all components are of similar power.

**Figure 9:**
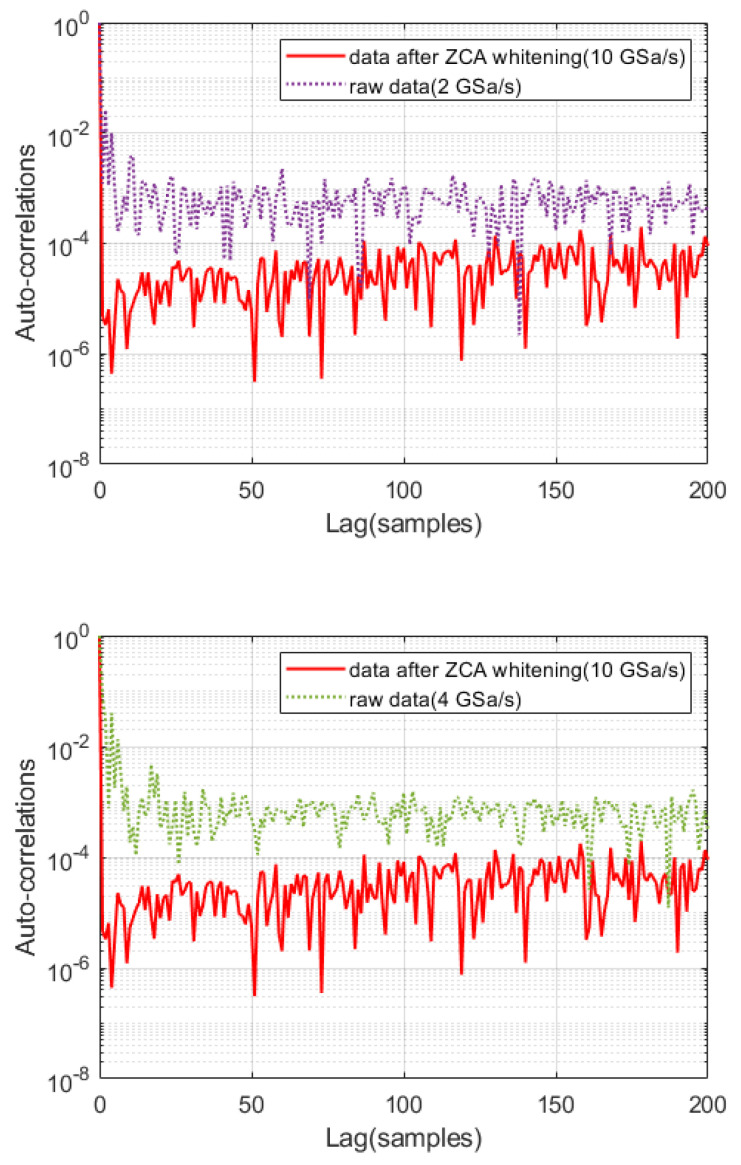
Auto-correlation coefficients of raw data2 and raw data3.

To further validate the effectiveness of our method, the raw data under the sampling rate of 4 GSa/s and 2 GSa/s is, respectively, acquired, and the auto-correlation coefficient is calculated, which are compared with the auto-correlation coefficient of the data after ZCA whitening under the sampling rate of 10 GSa/s, as shown in Figure 9, demonstrating that our method can effectively reduce the auto-correlation coefficient. To acquire the final quantum random number, the entropy evaluation and randomness extraction is performed on the raw data under the sampling rate of 4 GSa/s and 2 GSa/s. Compared to the generation rate of 40 Gbps based on ZCA whitening, the sampling rate of 4 GSa/s and 2 GSa/s lead to the generation rates of only 16 Gbps and 32 Gbps, respectively, further indicating that our method enhance the random number generation rate of QRNG.

## 4. Conclusions

In this work, a novel post-processing method of QRNG based on ZCA whitening is proposed and theoretically analyzed. The significance of this new method is that, by employing the ZCA whitening on the raw data, the statistical characteristics of the processed data will be more random, and the generation rate of the QRNG can be improved. However, there are several limitations to this work need to be further considered, such as the sufficient computing power for matrix factorization and more detailed digital signal processing analysis. Future research is needed to improve this simple but effective method.

## Data Availability

The data are available upon request from the corresponding author.
